# Effects of antibiotic therapy on the early development of gut microbiota and butyrate-producers in early infants

**DOI:** 10.3389/fmicb.2024.1508217

**Published:** 2025-01-07

**Authors:** Jun Qiu, Sha Wu, Ruiwen Huang, Zhenyu Liao, Xiongfeng Pan, Kunyan Zhao, Yunlong Peng, Shiting Xiang, Yunhui Cao, Ye Ma, Zhenghui Xiao

**Affiliations:** ^1^The School of Pediatrics, Hengyang Medical School, University of South China, Hunan Children’s Hospital, Hengyang, Hunan, China; ^2^Pediatrics Research Institute of Hunan Province, Hunan Children's Hospital, Changsha, Hunan, China; ^3^Department of Neonatology, Hunan Children's Hospital, Changsha, Hunan, China; ^4^The School of Public Health, University of South China, Hengyang, China; ^5^Department of Epidemiology and Health Statistics, Medical College of Soochow University, Suzhou, China; ^6^Department of Emergency Center, Hunan Children’s Hospital, Changsha, China

**Keywords:** gut microbiota, antibiotics, amoxicillin-clavulanic acid, moxalactam, butyrate-producers

## Abstract

**Background:**

Antibiotics, as the most commonly prescribed class of drugs in neonatal intensive care units, have an important impact on the developing neonatal gut microbiota. Therefore, comprehending the effects of commonly used antibiotic therapy on the gut microbiota and butyrate-producers in early infants could provide information for therapeutic decision-making in the NICU.

**Objectives:**

To explore the effects of antibiotic therapy on the early development of gut microbiota and butyrate-producers in early infants.

**Methods:**

A total of 72 infants were included in the study. We performed 16S rRNA sequencing on stool swab samples collected from neonatal intensive care unit patients who received amoxicillin-clavulanic acid (AC, *n* = 10), moxalactam (ML, *n* = 28) and non-antibiotics (NA, *n* = 34). We then compared the taxonomic composition between treatment regimens, focusing on differences in butyrate-producers.

**Results:**

Our study showed that there were significant differences in Shannon index (*p* = 0.033) and Beta diversity (*p* = 0.014) among the three groups. At the family level, compared with the other two groups, the relative abundance of *Clostridiaceae* (*p* < 0.001) and *Veillonellaceae* (*p* = 0.004) were significantly higher, while the relative abundance of *Enterococcidae* (*p* < 0.001) was significantly lower in the NA group. The relative abundance of *Enterobacteriaceae* (*p* = 0.022) in the AC group was greater than that in the other two groups. Additionally, butyrate-producers (*p* < 0.001), especially *Clostridiaceae* (*p* < 0.001), were noticeably more abundant in the NA group. The relative abundance of *Clostridiaceae* and butyrate-producers were the lowest in the ML group (*p* < 0.001).

**Conclusion:**

We found that antibiotic therapy had an adverse impact on the initial development of gut microbiota and leaded to a reduction in the abundance of butyrate-producers, particularly *Clostridiaceae*. Furthermore, moxalactam had a more pronounced effect on the gut microbiota compared to amoxicillin-clavulanic acid.

## Introduction

Antibiotics, as the most commonly prescribed class of drugs in neonatal intensive care units (NICUs), demonstrate dual effects. While antibiotics are lifesaving in treating infections, prolonged exposure (greater than 3 or 5 days) is associated with short-term complications, including necrotizing enterocolitis (NEC), late-onset sepsis (LOS), periventricular leukomalacia (PVL), retinopathy of prematurity (ROP), chronic lung disease (CLD), and death (Esaiassen et al., 2017; Ting et al., 2016; Carl et al., 2014). Similarly, this exposure is linked to an increased risk of long-term health outcomes such as obesity, inflammatory bowel disease and allergy (Ajslev et al., 2011; Örtqvist et al., 2019; Arrieta et al., 2015). Therefore, rational and standardized antibiotic usage is a crucial target for antimicrobial stewardship in NICUs.

Infancy is a critical period for the establishment and development of the gut microbiota, shaped not only by the gestational age, delivery mode, and feeding but also by medical interventions, such as antibiotics (Bokulich et al., 2016; Reyman et al., 2019). Antibiotic therapy can induce alterations in species diversity (alpha diversity) and community composition of the gut microbiota, with effects persisting for an extended period in children (Gasparrini et al., 2019). During this critical developmental window, perturbations in the gut microbiota can profoundly affect host physiology and disease risk (Cox et al., 2014; Vangay et al., 2015) including asthma (Yamamoto-Hanada et al., 2017), allergy (Kim et al., 2018), type 1 diabetes (Clausen et al., 2016), obesity (Block et al., 2018) and impaired neurocognitive outcomes (Slykerman et al., 2019).

Infancy also represents a pivotal time for acquiring butyrate-producers (Bokulich et al., 2016), whose metabolite butyrate plays a central role in metabolic functions (Zhang et al., 2021), acting as the primary energy source for colonocytes and exerting regulatory effects on local immune system homeostasis and glucose homeostasis (Zhang et al., 2021; Donohoe et al., 2011; Smith et al., 2013). Butyrate promotes intestinal barrier function, physiological mucosal hypoxia, and the proliferation of health-associated anaerobes (Peng et al., 2007; Kelly et al., 2015). Additionally, Butyrate exhibits protective effects in conditions such as obesity, type 2 diabetes, autism, and cardiovascular disease (Blaak et al., 2020; Liu et al., 2019; Chen et al., 2023). Given its crucial role in host health, understanding the communities that produce gut butyrate is a priority in gut microbiota research.

The research about the impacts of commonly used antibiotic therapies on gut microbiota and butyrate-producing bacteria in early infants remains limited. Therefore, we compared the effects of amoxicillin-clavulanic acid (AC), moxalactam (ML) and non-antibiotics (NA) on gut microbiota diversity, composition and butyrate-producing bacteria in NICU infants in the study. We aimed to explore the effects of antibiotic therapy on the early development of gut microbiota and butyrate-producers in early infants to inform treatment decisions and contribute to the standardization of antibiotic use in the NICU.

## Materials and methods

### Study design and participants

This neonatal cohort study was conducted at Hunan Children’s Hospital in China, from August 1, 2018, to October 31, 2019. Seventy-two infants were enrolled based on the administration and types of antibiotics they received, and were subsequently divided into three groups: the amoxicillin-clavulanic acid (AC) group, the moxalactam (ML) group, and the non-antibiotics (NA) group, comprising 10, 28, and 34 infants, respectively. Inclusion criteria included infants who had received at least one dose of AC or ML without concomitant use of other types of antibiotics or who had not received any antibiotics prior to stool swab sample collection. Exclusion criteria applied to infants lacking a stool swab sample or with documented prior exposure to different antibiotics prior to hospitalization. Additionally, infants who did not receive intravenous medications were excluded from the study. The flow chart is shown in Supplementary Figure S1. Ethical approval for this study was obtained from the Medical Ethics Committee of Hunan Children’s Hospital (HCHLL-2018-64), and informed consent was obtained from the parents of all participating infants.

### Butyrate-producers classification and quantification

According to a list of known butyrate-producers, we quantified the observed butyrate producers at the level of family, including *Clostridiaceae, Bacteroidaceae, Lachnospiraceae, Erysipelotrichaceae, Ruminococcaceae, Eubacteriaceae, and Fusobacteriaceae* (Rooney et al., 2020; Romick-Rosendale et al., 2018; Fu et al., 2019). Subsequently, we quantified the relative abundance and richness of these butyrate-producers in each sample.

### Gut microbiome analyses

Freshly evacuated fecal samples were collected into sterile tubes and immediately frozen in ice boxes, and transported to the laboratory within 2 hours. All samples were stored at −80°C until further processing. Bacterial DNA was extracted from the fecal samples using the QIAamp FAST DNA Stool Mini-Kit according to the manufacturer’s instructions. The V3-V4 region of the 16S rRNA gene was amplified using 341F/806R primers (341F: 5′-GTGCCAGCMGCCGCGG-3′/806R: 5′-GGACTACVVGGGTATCTAATC-3′), and polymerase chain reaction (PCR) was conducted. The amplified DNA was then sequenced using the Illumina MiSeq platform.

To ensure the accuracy in subsequent analyses, the raw reads were filtered based on the following criteria: reads containing bases with a terminal mass less than 20 and sequences shorter than 100 base pairs were discarded using Trim Galore software. The merged sequences were assembled using FLASH2 software. Primer sequences were removed using mothur software, and sequences exhibiting a base mismatch rate greater than 2% and shorter than 100 base pairs were eliminated using usearch, resulting in the acquisition of optimized sequences of high quality and reliability. The filtered sequences were then clustered into operational taxonomic units (OTUs) with a similarity threshold of ≥97%.

### Statistical analyses

Descriptive statistics were computed for the basic information of the study subjects. Normally distributed data were presented as mean ± standard deviation (X ± SD), while non-normally distributed data were expressed as median and interquartile range [M (P25–P75)]. Categorical data were described by absolute numbers. Group differences were assessed using the Chi-square test or Fisher’s exact test for categorical variables and the Kruskal-Wallis Test for continuous variables. Pairwise comparisons were conducted using the Wilcoxon rank-sum test. The alpha diversity index of the gut microbiota was compared among the three groups using R software, with significance assessed via the Kruskal-Wallis H rank sum test. The beta diversity was analyzed through principal coordinates analysis (PCoA) to demonstrate differences in gut microbiota composition among groups, with significance determined using the PERMANOVA-test. Linear discriminant analysis effect size (LEfSe) was used to identify the species most likely to explain differences between groups with the linear discriminant analysis (LDA).

Subjects were divided into three groups based on the presence and type of antibiotics, and the crude trends of gut microbiota composition were observed at the phylum, class, order, and family levels. Differences at these taxonomic levels and in butyrate-producers were analyzed using R software and IBM SPSS 27.0. Statistical analyses and mapping were conducted using IBM SPSS 27.0 and R 4.3.2. A *p*-value <0.05 was considered statistically significant.

## Results

### Clinical characteristics of the neonates

A total of 72 NICU infants were included in this study. The clinical characteristics of the neonates are presented in Table 1. There were no significant differences among the three groups in terms of gestational age, birth weight, day of age, sex ratio, mode of delivery, and type of feeding (*p* > 0.05).

**Table 1:** Comparison of basic information and clinical data of study subjects.

Clinical information	AC (*n* = 10)	ML (*n* = 28)	NA (*n* = 34)	*F*/*H*/*X*^2^	*p* value
Gestational Age* (weeks)	35.8 (33.9, 36.8)	37.9 (36.3, 39)	37.6 (35.7, 39.1)	5.259	0.072
Birth weight, mean (SD) (g)	2,441 ± 440	3,003 ± 741	2,812 ± 632	2.73	0.072
Day of age* (d)	20 (5, 42)	17 (10, 27)	9 (4, 26)	3.475	0.176
Male/Female, *n*	6/4	18/10	27/7	2.391	0.303
Vaginal/Cesarean, *n*	3/7	14/14	19/15	2.071	0.355
MBM/Non-MBM, *n*	3/7	14/14	6/28	2.697	0.260

AC, amoxicillin-clavulanic acid; ML, moxalactam; NA, non-antibiotics; SD, standard deviation; MBM, mother’s breastmilk.

*Median (P25-P75).

### Gut microbiota characteristics of the AC group, ML group, and NA group

**Figure 1:**
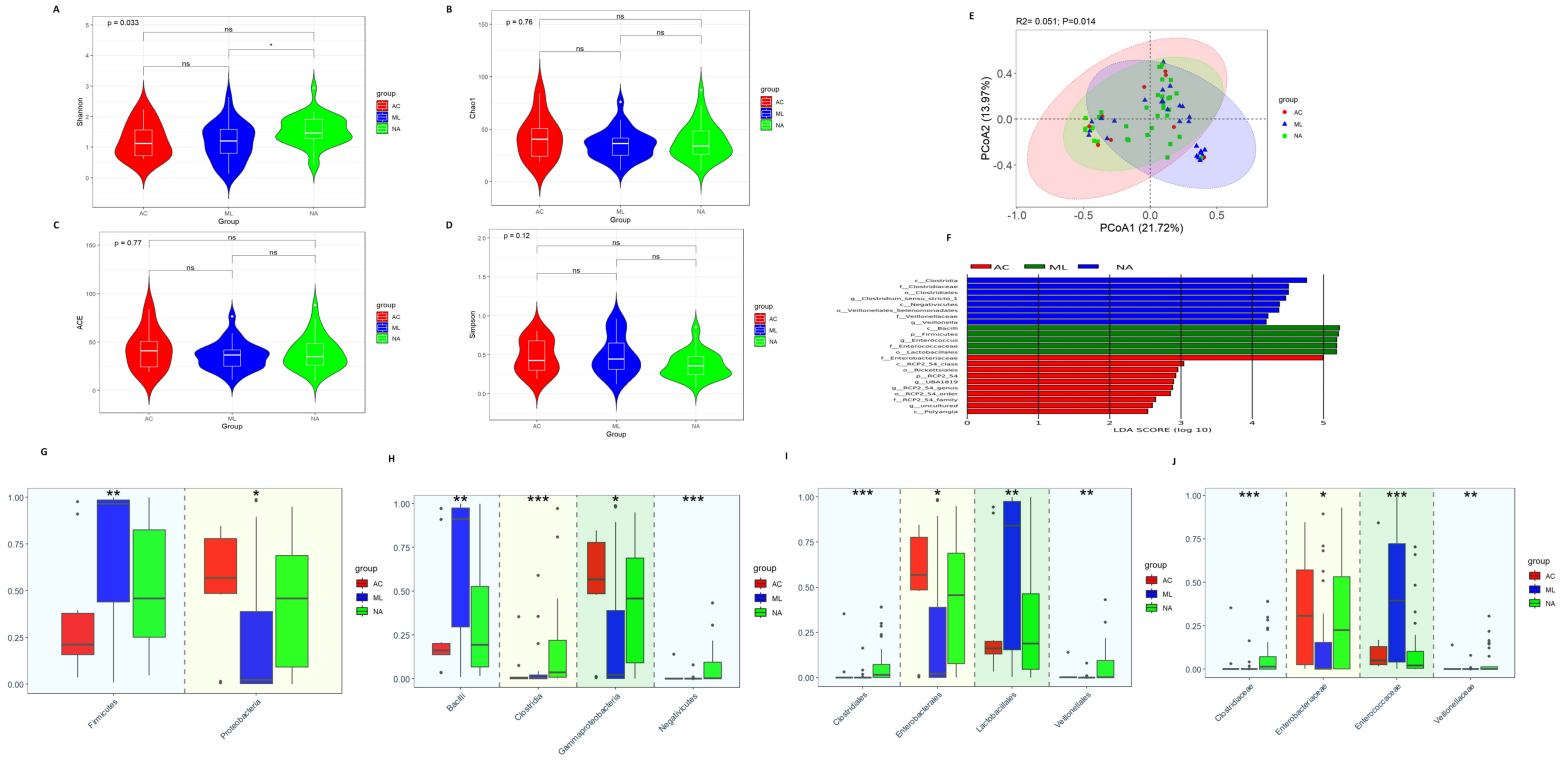
Gut microbiota diversity and relative abundance in the AC, ML, and NA groups. **(A)** Comparison of Shannon index among the three groups. **(B)** Comparison of Chao1 index among the three groups. **(C)** Comparison of ACE index among the three groups. **(D)** Comparison of Simpson index among the three groups. **(E)** PCoA among the three groups. **(F)** Lefse analysis among the three groups. **(G–J)** Differential bacteria in relative abundance among the three groups at the phylum, class, order and family level, respectively. *Indicates *p* < 0.05, ** Indicates *p* < 0.01, *** Indicates *p* < 0.001. AC, amoxicillin-clavulanic acid group; ML, moxalactam group. NA, non-antibiotics group.

Alpha diversity analysis found that the Shannon index (Figure 1A, 1.465vs. 1.125 vs.1.206 *p* = 0.033) was higher in the NA group than that in the AC group and ML group. However, there were no significant differences in Chao1, ACE, and Simpson index among the three groups (Figures 1B–D, *p* > 0.05). Differences in beta diversity were discovered among treatment regimens (Figure 1E, *p* = 0.014).

The LEfSe analysis (Figure 1F) showed eight bacteria (p, c, o, f, and g, respectively, representing phylum, class, order, family, and genus level) were enriched in the NA group, five bacteria were enriched in the ML group and ten bacteria were enriched in the AC group. *Clostridiaceae*, *Clostridium_sensu_stricto_1*, *Veillonellaceae*, and *Veillonella* were abundant in the NA group. *Firmicutes*, *Enterococcaceae*, and *Enterococcus* were abundant in the ML group. As well as, *Enterobacteriaceae* were abundant in the AC group.

At the phylum level (Figure 1G), there was increasing trend in the relative abundance of *Firmicutes* (0.212 vs. 0.459 vs. 0.965, *p* = 0.010) in the AC, NA and ML groups, while there was decreasing trend in the relative abundance of *Proteobacteria* (0.568 vs. 0.458 vs. 0.022, *p* = 0.025). At the class level (Figure 1H), there was increasing trend in the relative abundance of *Bacilli* (0.161 vs. 0.193 vs.0.911, *p* = 0.002) in the AC, NA and ML groups, while there was decreasing trend in the relative abundance of *Gammaproteobacteria* (0.567 vs. 0.458 vs. 0.022, *p* = 0.025). There was increasing trend in the relative abundance of *Clostridia* (0.002 vs. 0.003 vs. 0.036, *p* < 0.001) in the AC, ML and NA groups, while there was decreasing trend in the relative abundance of *Negativicutes* (0.002 vs. 0.0005 vs. 0.00006, *p* < 0.001). At the order level (Figure 1I), there was increasing trend in the relative abundance of *Lactobacillales* (0.161 vs. 0.188 vs. 0.841, *p* = 0.009) in the AC, NA and ML groups, while there was decreasing trend in the relative abundance of *Enterobacterales* (0.567 vs. 0.455 vs. 0.021, *p* = 0.025). The relative abundances of *Clostridiales* (0.0000 vs. 0.0003 vs. 0.0145, *p* < 0.001) and *Veillonellales* (0.00006 vs. 0.0005 vs. 0.0016, *p* = 0.003) showed an increasing trend in the ML group, AC group, and NA groups. At the family level (Figure 1J), there was increasing trend in the relative abundance of *Clostridiaceae* (0.0000 vs. 0.0003 vs. 0.0145, *p* < 0.001) and *Veillonellaceae* (0.00005 vs. 0.00053 vs. 0.00129, *p* = 0.004) in the ML, AC and NA groups. The relative abundances of *Enterobacteriaceae* (0.003 vs. 0.226 vs. 0.307, *p* = 0.022) showed an increasing trend in the ML group, NA group, and AC groups, while the relative abundances of *Enterococcaceae* (0.395 vs. 0.050 vs. 0.022, *p* < 0.001) showed a decreasing trend in the ML group, AC group, and NA groups.

### Gut microbiota characteristics of the AC group and ML group

**Figure 2:**
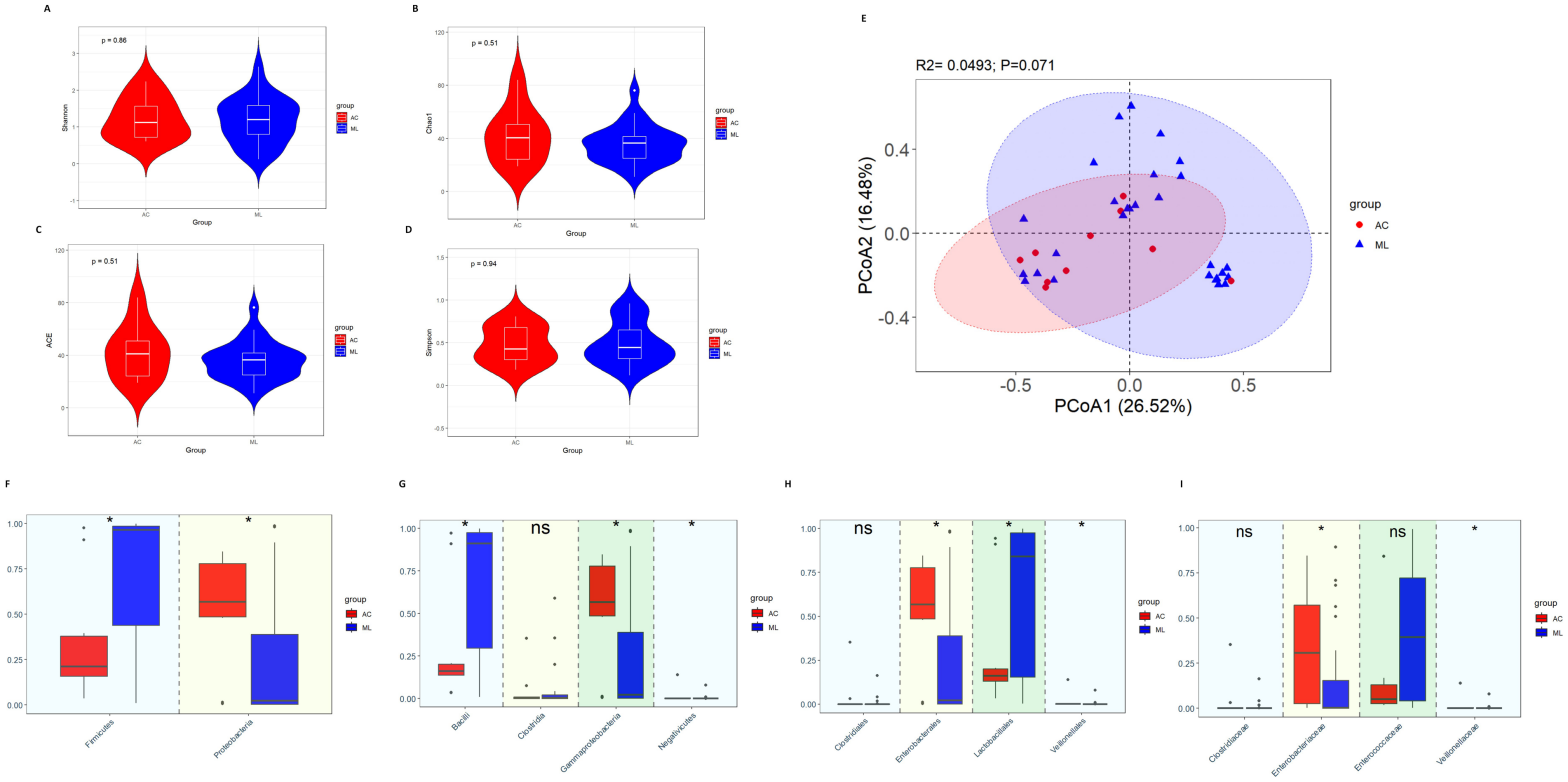
Gut microbiota diversity and relative abundance in the AC and ML groups. **(A)** Comparison of Shannon index between the two groups. **(B)** Comparison of Chao1 index between the two groups. **(C)** Comparison of ACE index between the two groups. **(D)** Comparison of Simpson index between the two groups. **(E)** PCoA between the two groups. **(F–I)** Differential bacteria in relative abundance between the two groups at the phylum, class, order and family level, respectively. *Indicates *p* < 0.05. AC, amoxicillin-clavulanic acid group; ML, moxalactam group.

There was nonsignificant difference in diversity between the AC group and ML group (Figures 2A–E).

Variations among the taxa were mainly compared at the phylum, class, order and family levels. At the phylum level (Figure 2F), compared with the ML group, the relative abundance of *Firmicutes* (0.212 vs. 0.965, *p* = 0.014) was significantly lower in the AC group, while Proteobacteria (0.568 vs. 0.022, *p* = 0.022) was significantly higher in the AC group. At the class level (Figure 2G), compared with the ML group, the relative abundance of Bacilli (0.161 vs. 0.911, *p* = 0.020) was significantly lower in the AC group, while *Gammaproteobacteria* (0.567 vs. 0.022, *p* = 0.022) and *Negativicutes* (0.0005 vs. 0.00006, *p* = 0.016) were significantly higher in the AC group. There was no difference in the relative abundance of *Clostridia* (0.002 vs. 0.003, *p* = 0.619) between the AC group and ML group. At the order level (Figure 2H), by comparing the two groups, the relative abundances of *Enterobacterales* (0.567 vs. 0.021, *p* = 0.026) and *Veillonellales* (0.0005 vs. 0.00006, *p* = 0.016) were noticeably greater in the AC group, while the abundances of *Lactobacillales* (0.161 vs. 0.841, *p* = 0.047) were significantly lower in the AC group. There was no difference in the relative abundance of *Clostridiales* (0.0003 vs. 0.0000, *p* = 0.066) between the AC group and ML group. At the family level (Figure 2I), by comparing the two groups, the relative abundances of *Enterobacteriaceae* (0.307 vs. 0.003, *p* = 0.017) and *Veillonellaceae* (0.00053 vs. 0.00005, *p* = 0.014) were noticeably greater in the AC group. There were no significant differences in the relative abundance of *Clostridiaceae* (0.0003 vs. 0.0000, *p* = 0.066) and *Enterococcaceae* (0.050 vs. 0.395, *p* = 0.085) between the AC group and ML group.

### Gut microbiota characteristics of the AC group and NA group

**Figure 3:**
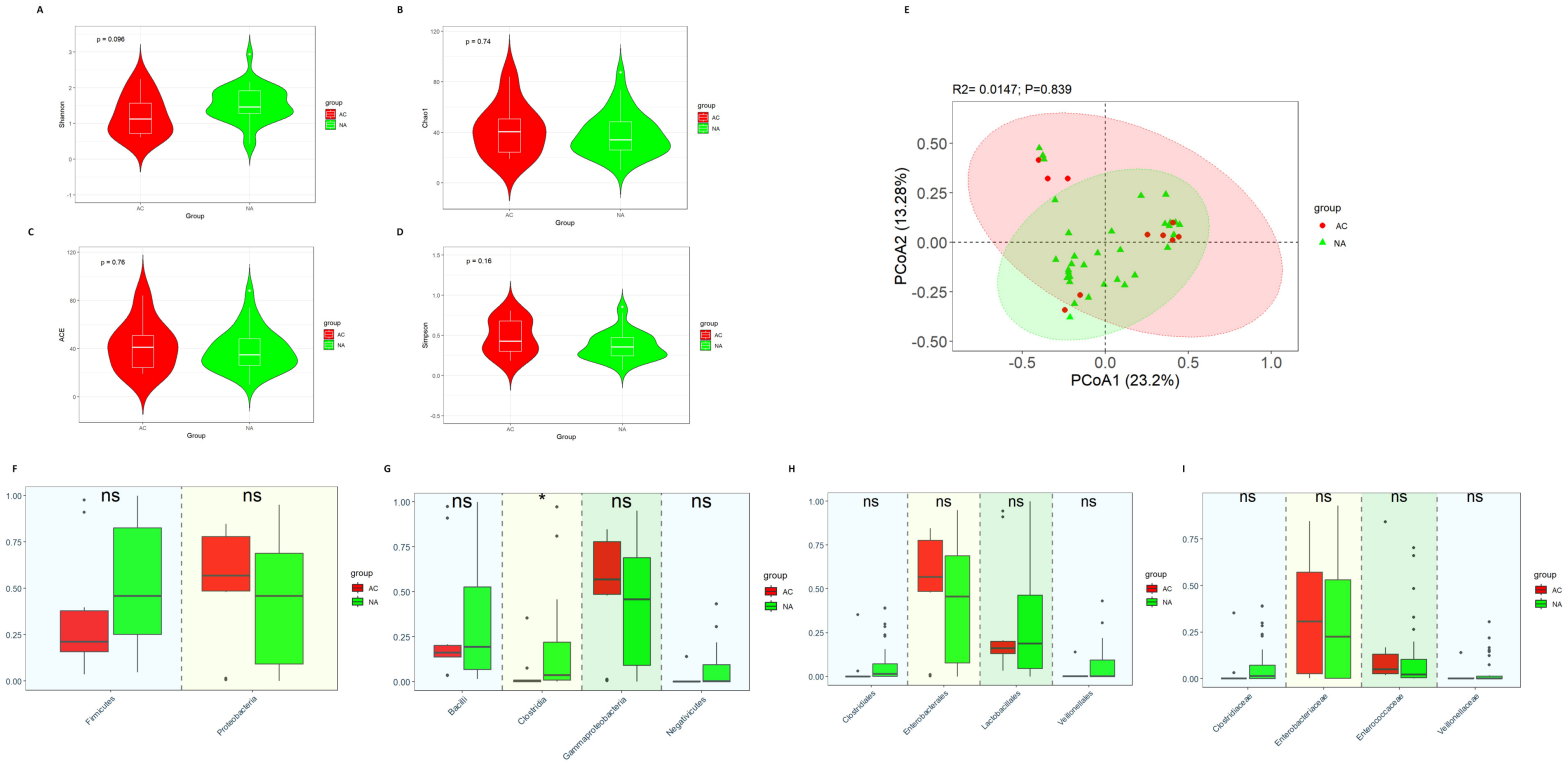
Gut microbiota diversity and relative abundance in the AC and NA groups. **(A)** Comparison of Shannon index between the two groups. **(B)** Comparison of Chao1 index between the two groups. **(C)** Comparison of ACE index between the two groups. **(D)** Comparison of Simpson index between the two groups. **(E)** PCoA between the two groups. **(F–I)** Differential bacteria in relative abundance between the two groups at the phylum, class, order and family level, respectively. *Indicates *p* < 0.05. AC, amoxicillin-clavulanic acid group; NA, non-antibiotics group.

There was no difference in diversity between the AC group and NA group (Figures 3A–E).

The results showed that there were no significant differences in the relative abundance of *Firmicutes* (0.212 vs. 0.459, *p* = 0.145) and *Proteobacteria* (0.568 vs. 0.458, *p* = 0.287) at the phylum level (Figure 3F). In terms of class (Figure 3G), *Clostridia* (0.002 vs. 0.036, *p* = 0.013) was significantly more abundant in the NA group. However, the relative abundances of *Bacilli* (0.161 vs. 0.193, *p* = 0.845), *Gammaproteobacteria* (0.567 vs. 0.458, *p* = 0.025) and *Negativicutes* (0.0005 vs. 0.002, *p* = 0.299) did not show the significant differences between the two groups. At the order and family level (Figures 3H,I), we did not observe the significant differences in the relative abundance of *Lactobacillales* (0.161 vs. 0.188, *p* = 0.955), *Enterobacterales* (0.567 vs. 0.455, *p* = 0.287), *Clostridiales* (0.0003 vs. 0.0145, *p* = 0.123), *Veillonellales* (0.0005 vs. 0.001, *p* = 0.491), *Clostridiaceae* (0.0003 vs. 0.0145, *p* = 0.123), *Veillonellaceae* (0.00053 vs. 0.00129, *p* = 0.715), *Enterobacteriaceae* (0.307 vs. 0.226, *p* = 0.450) and *Enterococcaceae* (0.050 vs. 0.022, *p* = 0.123) between the AC and NA groups.

### Gut microbiota characteristics of the ML group and NA group

**Figure 4:**
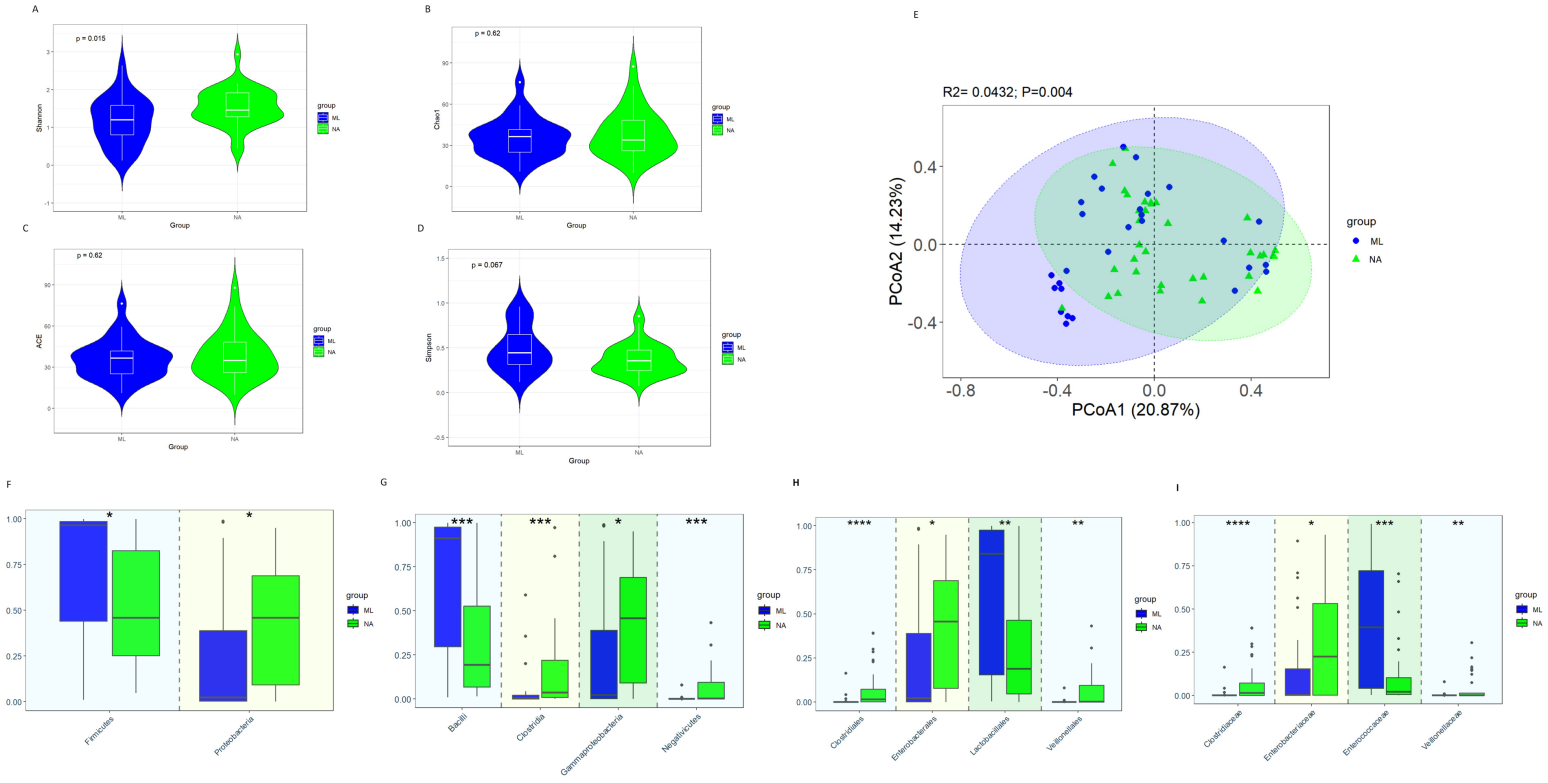
Gut microbiota diversity and relative abundance in the ML and NA groups. **(A)** Comparison of Shannon index between the two groups. **(B)** Comparison of Chao1 index between the two groups. **(C)** Comparison of ACE index between the two groups. **(D)** Comparison of Simpson index between the two groups. **(E)** PCoA between the two groups. **(F–I)** Differential bacteria in relative abundance between the two groups at the phylum, class, order and family level, respectively. *Indicates p < 0.05, ** Indicates *p* < 0.01, *** Indicates *p* < 0.001; ML, moxalactam group; NA, non-antibiotics group.

The alpha diversity showed that the Shannon index in the NA group was significantly higher than that in the ML group (Figure 4A, 1.46 vs. 1.21, *p* = 0.015). But there were no significant differences in the Chao1 index (Figure 4B, *p* = 0.62), ACE index (Figure 4C, *p* = 0.62), and Simpson index (Figure 4D, *p* = 0.067). The *β* diversity analysis (PCoA) results revealed significant differences in the intestinal microbiota between the ML group and the NA group (Figure 4E*, p* = 0.004).

At the phylum level (Figure 4F), when comparing the ML and NA groups, the relative abundance of *Firmicutes* (0.459 vs. 0.965, *p* = 0.017) was significantly less abundant in the NA group, while *Proteobacteria* (0.458 vs. 0.022, *p* = 0.033) was considerably greater in the NA group than in the ML group. At the class level (Figure 4G), *Gammaproteobacteria* (0.458 vs. 0.022, *p* = 0.033), *Clostridia* (0.036 vs. 0.003, *p* < 0.001), *Negativicutes* (0.002 vs. 0.00006, *p* < 0.001) were significantly more abundant in the NA group, while *Bacilli* (0.193 vs. 0.911, *p* < 0.001) were significantly less abundant in the NA group. At the order level (Figure 4H), *Enterobacterales* (0.455 vs. 0.021, *p* = 0.030), *Clostridiales* (0.0145 vs. 0.0000, *p* < 0.001) and *Veillonellales* (0.0016 vs. 0.00006, *p* = 0.001) were significantly more abundant in the NA group, while *Lactobacillales* (0.188 vs. 0.841, *p* = 0.004) was significantly less abundant in the NA group. At the family level (Figure 4I), the relative abundances of *Clostridiaceae* (0.0145 vs. 0.0000, *p* < 0.001), *Veillonellaceae* (0.00129 vs. 0.00005, *p* = 0.002) and *Enterobacteriaceae* (0.226 vs. 0.003, *p* = 0.026) were noticeably greater in the NA group, while the relative abundances of *Enterococcaceae* (0.022 vs. 0.395, *p* < 0.001) was significantly lower in the NA group.

### Butyrate-producers characteristics of the AC group, ML group, and NA group

**Figure 5:**
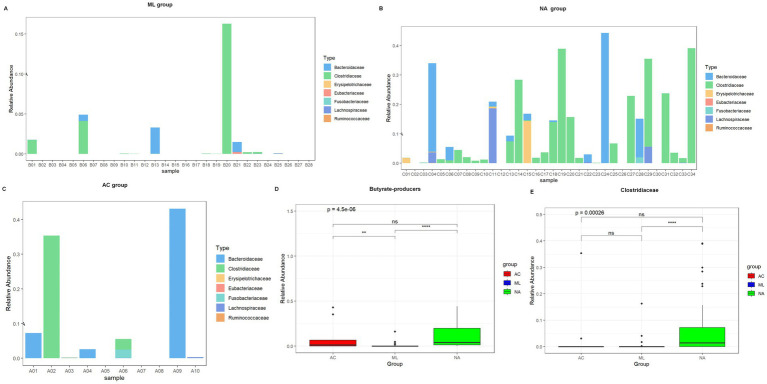
Relative abundance of butyrate-producers in three groups of infants, stratified by treatment regimen received: AC, ML, or NA. Relative abundance of butyrate-producers in ML group **(A)**. Relative abundance of butyrate-producers in NA group **(B)**. Relative abundance of butyrate-producers in AC group **(C)**. Total butyrate-producers content in three groups **(D)**. Relative abundance of Clostridiaceae in three groups **(E)**.

Compared with the AC group and ML group, the relative abundance of butyrate-producers (Figures 5A–D, 0.0409 vs. 0.0148 vs. 0.0001, *p* = 4.5e-06), especially *Clostridiaceae* (Figure 5E, 0.01449 vs. 0.00025 vs. 0.00000, *p* = 0.00026) were noticeably more abundant in the NA group. Other butyrate-producers, such as *Erysipelotrichaceae*, *Eubacteriaceae*, *Fusobacteriaceae*, *Lachnospiraceae*, and *Bacteroidaceae* exhibited no significant differences among the three groups (Supplementary Figure S2). When comparing the AC and ML groups, butyrate-producers (0.0148 vs. 0.0001, *p* = 0.009) exhibited higher abundances in the AC group, as shown in Figure 5D, while *Clostridiaceae* (0.00025 vs. 0.00000, *p* = 0.066) exhibited no significant difference between the two groups, as shown in Figure 5E. When comparing the AC and NA groups, the abundances of butyrate-producers (0.0148 vs. 0.0409, *p* = 0.327) and *Clostridiaceae* (0.00025 vs. 0.01449, *p* = 0.123) exhibited no significant difference between the two groups. When comparing the ML and NA groups, the abundances of butyrate-producers (0.0001 vs. 0.0409, *p* < 0.001) and *Clostridiaceae* (0.00000 vs. 0.01449, *p* < 0.001) were significantly lower in the ML group than in the NA group, as shown in Figures 5D,E.

## Discussion

In our study, we found that antibiotic exposure was associated with a reduction in gut microbiota diversity. Furthermore, at the family level, we found that antibiotic treatment led to an increase in the abundance of *Enterococcaceae*, while simultaneously resulting in a decrease in the abundance of butyrate-producers particularly *Clostridiaceae*. Additionally, we observed that the gut microbiota composition in the NA and AC groups had a high degree of similarity, while the ML group differed from them, indicating that moxalactam had a greater impact on the gut microbiota compared to amoxicillin-clavulanic acid.

In this study, we found a significant decrease in Shannon diversity of the gut microbiota in infants treated with moxalactam compared with non-antibiotic infants, which was consistent with previous studies showing that moxalactam reduces Shannon diversity of the gut microbiota (Zhu et al., 2017). Spatz et al. reported that amoxicillin-clavulanic acid significantly reduced the bacterial alpha-diversity (Shannon index) (Spatz et al., 2023), we observed Shannon index was higher in the NA group than that in the AC group, although the differences was nonsignificant. McDonnell et al. also reported that childhood antibiotic exposure was associated with reductions in microbial community richness and diversity (McDonnell et al., 2021). Moreover, there were studies finding that a reduced diversity of gut microbiota has been associated with the occurrence of NEC (Wang et al., 2009), LOS (Mai et al., 2013), allergic disease (Bisgaard et al., 2011) and diabetes (Vatanen et al., 2018) later in life. Therefore, antibiotics may increase the risk of disease by reducing the diversity of the gut microbiota.

Our results showed that antibiotic treatment led to a reduction in the abundance of *Clostridiaceae* at the family level, aligning with a previous study by Yu et al., who reported that antibiotics inhibited the *Clostridiaceae* (Yu et al., 2022). Additionally, a previous animal experiment reported that antibiotics reduced the abundance of *Clostridium,* while supplementing with *Clostridium butyricum* prevented aggravated inflammation and the dysregulated immune response characterized by greater M2 polarization of pulmonary macrophages and decreased release of IFN-*γ* and IL-17 as well as increased IL-5 levels (Zhu et al., 2021). *Clostridiaceae* plays a vital role in producing butyrate (Fu et al., 2019; Vital et al., 2014), which can offer fuel sources for the host and maintains the gut barrier (Hung et al., 2022). A decrease in *Clostridiaceae* has been linked to conditions like diabetes (T1D) (Elhag et al., 2020; Chen et al., 2021), intestinal tumor (Chen et al., 2020), and neurodevelopmental disorders (Hung et al., 2022). Our study found that antibiotic treatment led to an increased abundance of *Enterococcaceae*, which is consistent with a previous study (Yu et al., 2018). Moreover, Reyman et al. reported that antibiotics also increased the abundance of Enterococcus (Reyman et al., 2022), which were pathogenic bacteria widely recognized as leading hospital pathogens. Enterococcus could cause cell or organ damage by secreting proteins and producing toxic oxygen metabolites. Additionally, they exhibited a propensity for developing resistance and were associated with various conditions, including bacteremia, intra-abdominal infections, endocarditis, as well as inflammatory bowel diseases (Fiore et al., 2019). Consequently, antibiotics increase the risk of disease by altering the ratio of beneficial to pathogenic bacteria, disrupting the balance between microbial communities.

Both types of antibiotic treatment involved in this study led to a decrease of butyrate-producers, which suggested that antibiotics inhibited the production of butyrate-producing bacteria. This observation is consistent with the previous studies, which found antibiotics have a specific and pronounced negative effect on butyrate production in the gut (Zaura et al., 2015). In addition, Rooney et al. found that each additional day of antibiotics was associated with lower richness of butyrate producers within a week after therapy (Rooney et al., 2020). Butyrate, a product of microbial fermentation of dietary fibers in the lower intestinal tract, plays an important role in the overall health (Canani et al., 2011). It supports intestinal homeostasis (Hamer et al., 2008), and improves inflammation, oxidative status, epithelial defense barriers, visceral sensitivity, and intestinal motility (Liu et al., 2018). Butyrate-producers are crucial in maintaining a healthy gut environment by limiting the colonization of pathogenic microbes (Singh et al., 2022), supporting an anaerobic environment in the gut to prevent pathogenic expansions like Salmonella and *E. coli* (Rivera-Chávez et al., 2016; Byndloss et al., 2017), and contributing to vitamin biosynthesis, particularly vitamin B complexes (Belzer et al., 2017). Depletion of butyrate-producing bacteria has been associated with several non-communicable diseases, such as type 2 diabetes mellitus (T2D) (Qin et al., 2012), obesity (Le Chatelier et al., 2013), and cardiovascular disease (Chen et al., 2023), as well as an increased risk of intestinal pathogens due to disrupted colonization resistance (Vital et al., 2017). Therefore, the disruption of gut microbiota by antibiotics, which increases the risk of disease, may be associated with a reduction in butyrate-producing bacteria. Given the potential of butyrate-producers as next-generation probiotics, understanding the impact of antibiotics on these bacteria is crucial. Supplementation with butyrate-producing bacteria might help mitigate the effects of antibiotics on the gut microbiota.

Previous studies have suggested that early childhood exposure to antibiotics may have an impact on their long-term growth and development (Gerber et al., 2016). Antibiotics may affect growth and development by disrupting the gut microbiota (Korpela and de Vos, 2016). In animal experiment, butyrate-producing bacteria have been proven to be important in improving the growth in aquatic animals (Liang et al., 2023). Concurrently, studies have shown that butyrate-producing bacteria can suppress fat accumulation, thereby reducing the risk of obesity (Asano et al., 2019). In this study, we found that antibiotics reduced the abundance of butyrate-producers, especially *Clostridiaceae*. Therefore, we next want to investigate the role that butyrate-producers play in modulating the effects of antibiotics on growth and development. Additionally, we aim to determine whether butyrate-producing bacteria could serve as a probiotic intervention to alleviate the impact of antibiotics on the gut microbiota.

Our study highlighted important findings about the relationship between antibiotic exposure and gut microbiota especially butyrate-producers in infant. A key strength of this study is the focus on the specific effects of individual antibiotics, such as amoxicillin-clavulanic acid and moxalactam, rather than overall antibiotic exposure. Additionally, we concentrated on examining the impact of these specific antibiotics on the abundance of butyrate-producing bacteria and revealed the differences in *Clostridiaceae*.

Our study has some limitations that should be acknowledged. Firstly, it was a cross-sectional, single-center study with a small sample size. Therefore, the results may not be generalizable to a larger population. Secondly, we had limited sampling time points, with only one time point analyzed. As a result, we were unable to determine the long-term effects of antibiotic treatments on gut microbiota. Finally, we conducted an initial analysis of the gut microbiota and identified disparities among various treatment groups by 16S rRNA sequencing. Nevertheless, the precise mechanisms through which antibiotics influence gut microbiota remain obscure. Consequently, subsequent research will be required to undertake animal and cellular experiments to elucidate the effects of antibiotics on the pathogenesis of gut microbiota.

## Conclusion

Infants with antibiotics treatment exhibit a reduction in gut microbiota diversity, a decrease in the relative abundance of butyrate-producers, especially Clostridiaceae, and an increase in the relative abundance of Enterococcidae. Indicating that antibiotic therapy has an adverse effect on the early development of gut microbiota and butyrate-producers, especially *Clostridiaceae* in Early Infants. Moreover, this study revealed moxalactam had a more pronounced influence on the gut microbiota compared to amoxicillin-clavulanic acid.

## Data Availability

The original contributions presented in the study are included in the article/supplementary material, further inquiries can be directed to the corresponding author.
