# Supplementary material for: Effects of antibiotic therapy on the early development of gut microbiota and butyrate-producers in early infants
Source: Front Microbiol. 2025 Jan 7;15:1508217. doi: 10.3389/fmicb.2024.1508217 (PMC11748296; doi:10.3389/fmicb.2024.1508217)
Supplement: Supplementary file 1 [file Presentation_1.pdf]

# Supplementary Materials

**Contents**

**Figure S1:** Flow chart of the participants included.

**Figure S2**: Relative abundance of other different butyrate-producers in three groups of infants.

Figure S1: Flow chart of the participants included

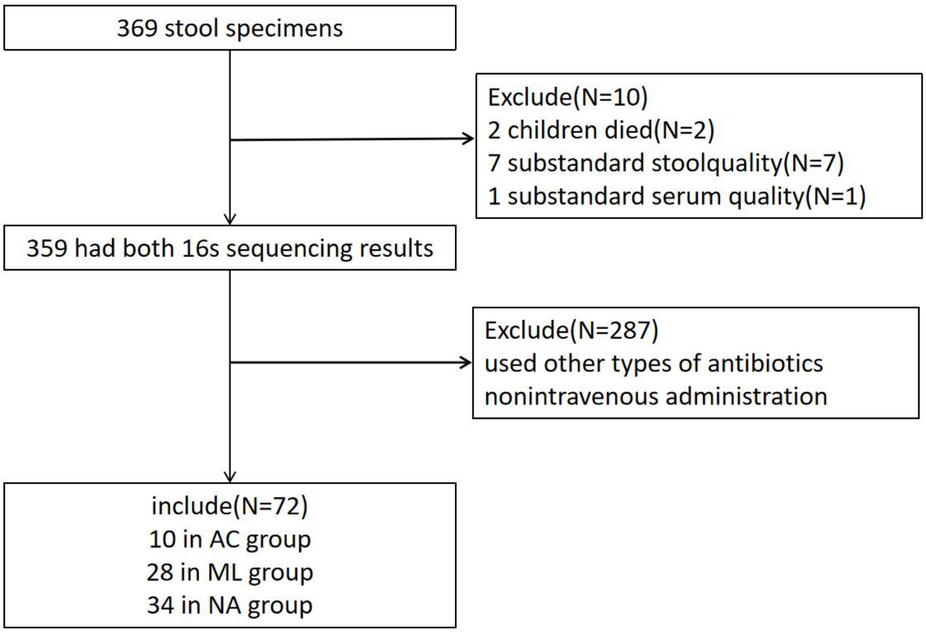

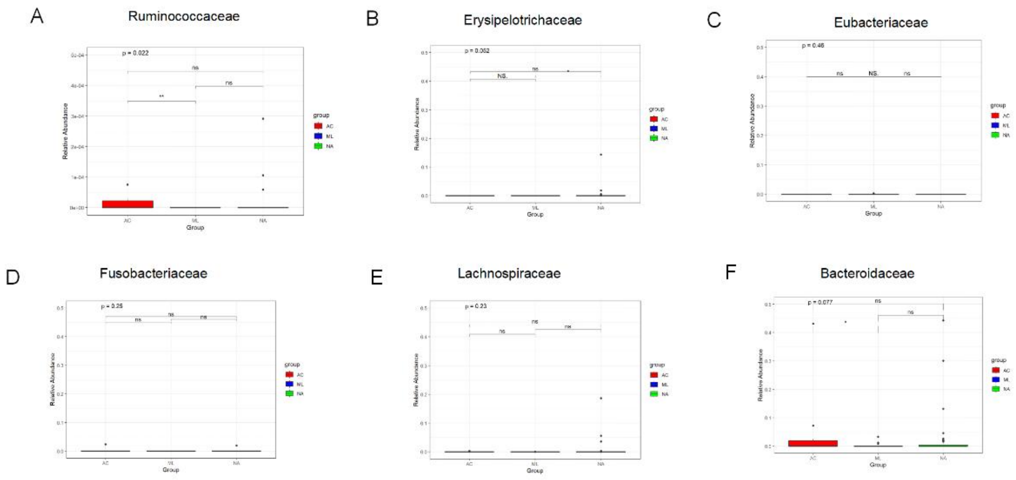


Figure S2. Relative abundance of other different butyrate-producers in three groups of infants. Relative abundance of *Ruminococcaceae* in three groups(A), Relative abundance of *Erysipelotrichaceae* in three groups(B); Relative abundance of *Eubacteriaceae* in three groups(C); Relative abundance of *Fusobacteriaceae* in three groups(D); Relative abundance of *Lachnospiraceae* in three groups(E); Relative abundance of *Bacteroidaceae* in three groups(F).
